# A New Window into Structural Plasticity in the Adult Visual Cortex

**DOI:** 10.1371/journal.pbio.0040042

**Published:** 2005-12-27

**Authors:** Liza Gross

The developing human brain is a hotbed of activity that continues well beyond the first year. During early postnatal development, we manufacture some 250,000 neurons per minute, then spend the next few years building the connections that underlie brain function. It has long been assumed that the neural plasticity of youth eventually settles down by adulthood. Though experimentally induced lesions in the adult cat and monkey cortex can produce anatomical changes, these findings are based on inferences from statistical evidence across different populations rather than on direct observation. And while neuroscientists have known for decades that the adult brain can reorganize neural pathways in response to new experiences—by changing the firing pattern and responses of neurons, for example—it has remained an open question whether structural changes accompany this functional plasticity.

In a new study, Wei-Chung Allen Lee and Elly Nedivi, along with Hayden Huang and Peter So, and their colleagues, take advantage of recent advances in imaging technology and single-cell genetic labeling techniques to investigate this question in mice. Continuous observations of the mouse adult visual cortex over the course of a few months revealed that the adult brain can indeed rewire its circuits under normal conditions. These rearrangements appear to follow neuron-specific rules, with one type of neuron undergoing a range of structural modifications while another maintains its original architecture. [Fig pbio-0040042-g001]

**Figure pbio-0040042-g001:**
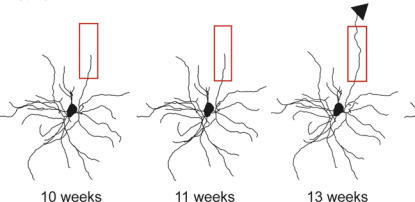
One of the dendritic branch tips of this inhibitory neuron grew out of the microscope's imaging area in just two weeks

Many studies have focused on pyramidal neurons—excitatory neurons that promote neuron firing—but few have focused on the possible structural dynamics of a range of different neuron types. In this study, Lee et al. focused on a cross-section of neurons, imaging every neuron they saw. The only neurons they saw growing were the inhibitory, nonpyramidal neurons, which inhibit the activity of cortical neurons and lack the classic pyramid structure that so easily identifies their excitatory brethren. Since these neurons can help adjust the brain's internal maps by inhibiting signaling in response to new stimuli or learning, the authors wondered if they could be involved in structural changes as well.

The authors focused on the surface layers of the neocortex. (The neocortex consists of six cell layers, with layer 1 closest to the cortical surface; the authors focused on layers 2 and 3.) To allow direct observation of the area, they implanted a glass window over the two areas of the visual cortex in four- to six-week-old mice. These mice express fluorescent protein in neocortical neurons, allowing Lee et al. to track the location and morphology of these neurons using two-photon microscopy. Time-lapse images of six pyramidal neurons and eight nonpyramidal neurons in 13 mice were taken over the course of four to ten weeks. The length of dendritic branch tips were measured over time to evaluate physical changes in the neurons.

The pyramidal neurons showed no structural changes in individual branch tips, but the nonpyramidal neurons showed dynamic changes, with one branch tip undergoing dramatic remodeling. “Within as little as two weeks,” the authors note, “this branch tip more than doubled its length and exited the imaging volume.” One nonpyramidal neuron even showed a few new branch tip additions.

All of the nonpyramidal neurons showed at least one and up to seven dynamic branch tips, with an average of about 14% showing structural modifications. (The authors monitored up to 50 branch tips from a single neuron.) This remodeling occurred both incrementally and in short bursts, and involved both branch tip growth and shrinking. Lee et al. confirmed that these nonpyramidal neurons were in fact inhibitory interneurons by showing that they expressed gamma-aminobutyric acid (GABA)—a neurotransmitter that inhibits neuron firing—while the pyramidal neurons did not.

Since the laws of probability suggest that given the changes observed in the nonpyramidal neurons, at least one pyramidal branch tip of 124 monitored should change if all things are equal, the authors argue that the pyramidal and nonpyramidal neurons have different dynamic properties. The branch tips of nonpyramidal cells in the adult neocortex can grow, retract, and sprout new additions—without experimental manipulations. Many studies support the idea of a relatively stable adult neocortical structure, but as Lee et al. point out, they focused on pyramidal neurons, while this study focused on nonpyramidal neurons. Both may be right. Under normal conditions at least, pyramidal structural modifications are far less obvious than those seen in the nonpyramidal neurons.

It remains to be seen whether the structural plasticity seen here underlies observed functional reorganizations. Probing this question will depend on determining what kinds of structural changes might be expected, figuring out how to detect them, and then interpreting the changes. Studying the responses of axonal arbors connected to the nonpyramidal dendrites, for example, may prove instructive. Based on these results, direct observation of specific neurons in a local pathway should yield promising results.

